# Addressing the ethical and legal complexities of hospital visitation restrictions for patients who are incarcerated

**DOI:** 10.1002/jhm.70022

**Published:** 2025-03-10

**Authors:** Amber R. Comer

**Affiliations:** ^1^ American Medical Association, Indiana University Indianapolis Indiana USA

## INTRODUCTION

Hospitalization often has a negative effect on patients’ emotional and psychological wellbeing with many patients experiencing loneliness, anxiety, and depression during their stay.^1^, ^2^, ^3^ While most hospitalized patients are uncomfortable, patients who are incarcerated often face security protocols, such as being handcuffed to the hospital bed, which contributes to making their hospitalization even more daunting.^4^ One security protocol which elicits complex legal and ethical dilemmas is restriction of hospital visitation with patients who are incarcerated. Given that over 7.6 million people are admitted to jail each year in the United States and that the population is aging and experiencing chronic health conditions increasing their likelihood of hospitalization, addressing ethical concerns within visitation policies for hospitalized patients who are incarcerated is imperative.^5^

Visitation restrictions, despite their rationale, are complex because having visitors during hospitalization is a vital part of recovery and emotional well‐being. For example, hospital visitor restrictions have been associated with the increased likelihood of delirium, loneliness, and discordant medical decisions for patients, as well as increased moral distress and ethical dilemmas for clinical care providers.^6^, ^7^ In addition to potential negative emotional effects, restrictions on hospital visitation can hinder patient autonomy and stifle shared medical decision making when a patient lacks capacity due to a breakdown of communication between the patient's surrogate medical decision maker and the clinical care team.^7^, ^8^, ^9^

Despite the potential negative effects of hospital visitor restrictions, restrictions are sometimes necessary to curb the spread of infectious diseases, for example, during a viral epidemic within the community.^10^, ^11^ While restrictions to hospital visitors are ethically and legally acceptable when implemented as a precaution for protecting patient and staff safety, visitation policies for patients who are incarcerated circumscribe the visitor policies in place for the general patient population, leaving incarcerated patients vulnerable to persistent visitor restrictions during their hospitalization.^12^ This article explores the ethical and legal complexities of hospital visitation restrictions for patients who are incarcerated and provides recommendations for visitation policies for this vulnerable patient population.

## THE LAW DRIVING HOSPITAL VISITATION RESTRICTIONS FOR PATIENTS WHO ARE INCARCERATED

Visitation policies for patients who are incarcerated are usually distinct from visitor policies in place for the general patient population because the law and ethics guiding visitation policies with prisoners is a balance between the objectives of the penal system and the rights of prisoners.^7^, ^8^, ^9^, ^11^, ^12^, ^13^ Penal system visitation policies are driven by a need to protect the hospital staff and the public by preventing patients who are incarcerated from gaining access to weapons, contraband, or a means to escape. Often, maintaining the security of patients who are incarcerated is reliant on a sole prison guard placed at the patient's door and restraints placed on the patient's wrist. These security concerns are further heightened when visitation is permitted.

Correctional facilities under federal jurisdiction follow federal regulations which only guarantee hospital visitation for incarcerated patients who have been diagnosed with a terminal illness.^14^ Sans a terminal diagnosis, the federal regulations allow individual hospitals to set their own visitation policies for patients who are incarcerated with the caveat that visitation may be restricted to immediate family members.^15^ While there is a regulation which applies to federal correctional facilities, there is no federal law or regulation which pertains to individual state systems; therefore, each state provides its own guidance for hospital visitation with patients who are incarcerated under their jurisdiction.^11^ The only federal guidance in this issue is a federal regulation that all hospitals which accept Medicare and Medicaid must have written policies and procedures regarding patient visitation.^16^ The absence of formal guidance on visitation with patients who are incarcerated has led to widespread disparate visitation policies throughout the nation.

While individual hospitals are responsible for setting and enforcing general patient visitation policies, the policies which guide visitation for a patient who is incarcerated come from two sources: the hospital in which the patient is admitted and the carceral system of which the patient is under the jurisdiction. Although there is no official law or regulation which requires hospitals to yield to the policies developed by the carceral system, this tends to be the default during clinical practice. Discordance between carceral jurisdiction and hospital visitation policies have significant potential to create ethical dilemmas when treating patients who are incarcerated.

## THE ETHICAL COMPLEXITIES OF RESTRICTING VISITATION WITH PATIENTS WHO ARE INCARCERATED

As there is not a right for incarcerated persons to be permitted visitors during hospitalization unless the patient is terminally ill, incarcerated patients are often completely restricted from having visitors. These burdensome restrictions have the effect of disconnecting the incarcerated person from their support system during a time which is known to be emotionally and psychologically taxing.

While there are some regulations pertaining to hospital visitation for incarcerated patients, the few which exist are ethically challenging. First, most policies (including federal regulations) only allow incarcerated patients to have visitors when they are terminally ill. Because it is often difficult to predict individual mortality, patients who are incarcerated may become incapacitated or die before they are able to have visitors, thus denying the patient the opportunity to say goodbye to their loved ones.^17^

Even when terminally ill patients who are incarcerated are permitted to have visitors during their hospitalization, the visitor may be forbidden from engaging in physical contact with the patient.^18^ The effect of this policy is that a parent may be forbidden from holding the hand of their dying child, regardless of the magnitude of the crime in which they are incarcerated, just because he was under the custody of the carceral system while dying. The no contact policy while visiting hospitalized incarcerated patients is, in some ways, rather ironic because in some states, prisoners on death row are allowed one final contact visit before their execution. For example, in California, prisoners on death row are permitted a final contact visit.^18^

An additional reason that only allowing terminally ill patients to have visitors is too narrow of a restriction is that this policy denies patients who may not be critically ill but are still facing the possibility of death an opportunity for support. For example, a patient who is poised to undergo a risky operation may not meet the definition of terminally ill, even though there may be a high likelihood of severe complications or death during the operation. Denying a patient facing a complicated and/or risky surgery visit may increase anxiety for the patient and can deny the patient the opportunity to gain support and/or say goodbye to their loved one.

Another ethical complication with visitation policies (including federal visitation regulations) is that many of these policies restrict visitors to immediate family members only. Restricting policies to only immediate family members may exclude the patient's main support person or persons from visiting. For example, many states do not recognize common law marriage, and as such, regardless of how long a couple has been together, unmarried partners are not legally considered immediate family members. Additionally, many families do not necessarily consist of the traditional nuclear family; thus, these restrictions could, in effect, leave the patient “unbefriended” or in a position where they have no qualified visitors.^19^ For example, a grandmother who raised her grandson may be precluded from visiting him in the hospital because grandparents are not usually considered immediate family members.

While visitation is important for emotional and psychological support of hospitalized patients, it is crucial for patients who lack capacity. When a patient lacks capacity, a surrogate is responsible for making their medical decisions. While this is a daunting task under any circumstance, visitation policies play a significant role in the process of surrogate medical decision making because in‐person visits allow the surrogate to communicate and form a relationship with the patient's clinical care team, as well as with the patient for whom they are making decisions.^20^, ^21^ The relationship between the surrogate and the physician or clinical care team is crucial for ensuring that the surrogate can uphold the patient's autonomy and be an active advocate for the patient.^22^ Visitation restrictions, which sometimes even include telephone conversations, of surrogate medical decision makers have been found to delay medical decision making, result in negative physical and mental health changes for the patient, and anxiety among surrogates.^23^, ^24^

Visitation restrictions become extremely complicated and ethically questionable when the incarcerated patient has legally appointed a surrogate medical decision maker that is not within what the state law considers their immediate family. Most visitation polices preclude non‐immediate family members from visitation and do not make an explicit exception for a patient's surrogate medical decision maker; therefore, surrogate medical decision makers outside of the patient's immediate family may not be able to visit during the patient's hospitalization.

In addition to visitation policies being restrictive, they also lack transparency and are subject to bias because most carceral policies require approval for visitation from the Watch Commander or some other representative of the carceral system such as the Warden.^17^ Without a clear process for how and why the Watch Commander approves visitation, patients who are incarcerated are left to the mercy of the Watch Commander to secure visitation.

## RECOMMENDATIONS FOR VISITATION POLICIES FOR HOSPITALIZED PATIENTS WHO ARE INCARCERATED

When considering hospital visitation policies for incarcerated patients, it is important to remember that incarcerated patients are human beings who experience loneliness, anxiety, and depression just like any other hospitalized patient. However, it is also imperative to address the safety and security concerns which arise when treating a patient who is incarcerated. While patients who are incarcerated pose potential safety and security concerns, hospitals are not strangers to addressing these types of threats. For example, some hospitalized patients experiencing mental health disorders pose similar safety and security concerns (i.e., the potential for escape, harm to staff, and access to harmful contraband) as patients who are incarcerated. To protect these patients, the staff, and the public, hospitals implement security measures such as locked units and supervised visitation.

Hospitals must balance the safety and security as well as comfort of their staff, the public, and the patient they are serving with the patient's increased wellbeing which is derived from visitation while also minimizing secondary gain that may be derived from being hospitalized during incarceration. Outside of hospitalization, regular visitation is permitted at the carceral facility, and this visitation may occur with people outside of the incarcerated person's immediate family. The narrow restrictions on hospital visitation, in some ways, serve as an ethical double jeopardy for patients who are incarcerated, functionally restricting the patient's support system during a time when they need more support, not less. While it is likely not possible to allow the same visitation policies within hospitals that is permitted within carceral facilities, visitation policies for patients who are incarcerated should be expanded to allow the following:

- Allow visitation for patients who are terminally ill, facing surgery, or who have a prolonged length of hospital stay.
- Allow the patient to identify a main support person or persons who are permitted to visit during hospital visitation.
- Allow the patient's surrogate medical decision maker visitation, even if this person is not within the patient's immediate family.
- Allow some form of physical contact if desired by the patient, for example, handholding.
- Carceral systems and hospitals should provide transparent visitation requirements and visitation request approval processes and include an appeal process which may allow case‐by‐case determinations for when the patient's prognosis, medical decisions, or treatment trajectory are complicated and do not fit within the standard vitiation requirements.

## CONCLUSIONS

There is a lack of standard and transparent visitation policies for hospitalized patients who are incarcerated. Additionally, the visitation policies that do exist are often restrictive and subject to bias. Given the emotional, physical, and medical necessity of visitation during hospitalization, not allowing patients who are incarnated visitation, in essence, punishes them for being hospitalized due to illness. The recommendations for visitation of patients who are incarcerated provided in this article provide guidance for setting ethical and just visitation policies that respect the patient, their surrogate, and the clinical care team while balancing the responsibilities of the carceral system.

## CONFLICT OF INTEREST STATEMENT

The author declares no conflicts of interest.
